# Life-Cycle Assessment of Alkali-Activated Materials Incorporating Industrial Byproducts

**DOI:** 10.3390/ma14092401

**Published:** 2021-05-05

**Authors:** Iman Faridmehr, Moncef L. Nehdi, Mehdi Nikoo, Ghasan Fahim Huseien, Togay Ozbakkaloglu

**Affiliations:** 1 Institute of Architecture and Construction, South Ural State University, Lenin Prospect 76, 454080 Chelyabinsk, Russia; faridmekhri@susu.ru; 2 Department of Civil and Environmental Engineering, Western University, London, ON N6A 5B9, Canada; 3 Young Researchers and Elite Club, Ahvaz Branch, Islamic Azad University, Ahvaz 61349-37333, Iran; m.nikoo@iauahvaz.ac.ir; 4 Department of Building, School of Design and Environment, National University of Singapore, Singapore 117566, Singapore; bdggfh@nus.edu.sg; 5 Ingram School of Engineering, Texas State University, San Marcos, TX 78666-78667, USA; togay.oz@txstate.edu

**Keywords:** sustainability, life cycle inventory, CO_2_ emissions, embodied energy, artificial neural network, industrial byproduct

## Abstract

Eco-friendly and sustainable materials that are cost-effective, while having a reduced carbon footprint and energy consumption, are in great demand by the construction industry worldwide. Accordingly, alkali-activated materials (AAM) composed primarily of industrial byproducts have emerged as more desirable alternatives to ordinary Portland cement (OPC)-based concrete. Hence, this study investigates the cradle-to-gate life-cycle assessment (LCA) of ternary blended alkali-activated mortars made with industrial byproducts. Moreover, the embodied energy (EE), which represents an important parameter in cradle-to-gate life-cycle analysis, was investigated for 42 AAM mixtures. The boundary of the cradle-to-gate system was extended to include the mechanical and durability properties of AAMs on the basis of performance criteria. Using the experimental test database thus developed, an optimized artificial neural network (ANN) combined with the cuckoo optimization algorithm (COA) was developed to estimate the CO_2_ emissions and EE of AAMs. Considering the lack of systematic research on the cradle-to-gate LCA of AAMs in the literature, the results of this research provide new insights into the assessment of the environmental impact of AAM made with industrial byproducts. The final weight and bias values of the AAN model can be used to design AAM mixtures with targeted mechanical properties and CO_2_ emission considering desired amounts of industrial byproduct utilization in the mixture.

## 1. Introduction

The ordinary Portland cement (OPC) industry is responsible for 5% to 7% of all Carbon dioxide (CO_2_) emissions generated by human activities [1]. Continuing cement production at the current rate may cause irreparable damage to global ecological systems. Thus, the development of eco-efficient alternatives to OPC is of utmost importance. Moreover, efficient industrial waste management and reducing the consumption of non-renewable natural resources are vital for sustainable development and cleaner ecosystems. Since the disposal of industrial waste materials is often associated with adverse environmental impact, a wide range of so-called “green” concrete and mortar mixtures incorporating industrial byproducts has been developed [2,3,4,5,6,7,8]. Since OPC is the primary concrete constituent responsible for CO_2_ emissions and embodied energy (EE), efforts have been made to fully or partially replace it with supplementary cementitious materials (SCMs). Industrial byproducts and agricultural wastes such as fly ash (FA), also known as pulverized fuel ash in the United Kingdom, which is acquired from coal-fired power plants, ground granulated blast furnace slag (GBFS), which is obtained by water or steam quenching of molten iron slag, a by-product of iron and steel-making from blast furnaces, waste ceramic powder (WCP) which is a byproduct of ceramic and construction industries, palm oil fly ash (POFA) that is produced from palm oil fibres, bunches and shells used as fuel for power generation in mills, rice husk ash, and sugarcane bagasse ash, etc., have been all considered as SCMs for full or partial replacement of OPC.

Alkali-activated paste, mortar, and concrete are manufactured using industrial byproducts that have demonstrated eco-efficient features, while achieving appropriate mechanical strength and durability. Generally, such mortars and concretes are prepared using starting source materials rich in silicon (Si), aluminium (Al) and calcium (Ca), along with alkaline activators, such as sodium silicate or/and sodium hydroxide. The compatible nature of C-(A)-S-H and N-A-S-H gels has a significant influence on the alkali-activated materials (AAMs) and alkaline solution activated alumina-silicate systems, wherein both products may be obtained compared to the C-S-H gel obtained with OPC. AAMs allow full replacement of OPC primarily using SCMs in their formulation, thus resulting in OPC-free concrete and mortar. Various previous studies on AAMs indicated excellent properties, such as high early strength, enhanced resistance to aggressive environments, and reduced environmental impact compared to cement mortar [9,10,11].

The most commonly used materials in AAM manufacturing are FA, metakaolin (MK), and GBFS, as reported in [12,13,14]. Although previous research has confirmed the excellent properties of FA and GBFS based-AAMs, the very nature of these industrial by-products implies varying mineralogical and chemical compositions, making the standardization process to reach desirable and consistent mechanical and durability properties difficult. Moreover, AAMs do not require the clinker manufacturing process needed for OPC at 1350–1450 °C, but are rather produced at relatively lower temperatures of 25–100 °C. This leads to a substantial reduction in CO_2_ emissions resulting from the decarbonization of limestone along with a decrease in the embodied energy (EE) needed for clinker production. Nevertheless, to ensure reliable mechanical properties and environmental benefits of AAMs, appropriate life-cycle assessment (LCA) is necessary. LCA is a reliable, standardized methodology to evaluate the environmental features of AMMs and to demonstrate rationally that they represent an effective and viable alternative to OPC. Considering that the pertinent results reported in the open literature remain conflicting, the environmental impact of AAMs remains controversial and open to debate [15,16,17,18].

LCA is a method of quantifying the environmental impacts associated with a given product, whereby researchers create an inventory of the resources used and pollutants that emanate from the product production and use [19,20,21]. Therefore, it is a rational and robust tool for assessing the ecological feasibility of incorporating recycled wastes and industrial by products into green concrete production. To investigate the environmental impact of concrete manufacturing, it is necessary to evaluate the entire life cycle, starting from the extraction of raw materials to the final waste disposal stage. Attention should be paid to the environmental features of OPC substitution with alternative industrial by products in view of sustainability considerations. Previous literature has primarily focused on the mechanical properties and durability of alternative binder materials in concrete, but did not generally consider comprehensive LCA to environmentally justify cement substitution, as reported in [22,23,24,25]. Therefore, adopting the LCA method for replacement of OPC concrete with eco-efficient alternatives is essential [18]. Specific parameters in green concrete and mortar design should consider obtaining adequate workability, mechanical strength, durability, cost, aesthetics, along with enhanced environmental footprint.

Accordingly, the present study investigates the compressive strength (CS), durability, as well as the environmental impact of alkali-activated mortars composed of industrial by products. CO_2_ emissions and EE, which represent fundamental parameters in the cradle-to-gate LCA, were investigated in detail for 42 ternary-blended AAM mixtures. Using the experimental test database thus generated, an optimized artificial neural network (ANN) combined with the cuckoo optimization algorithm was developed to estimate the CO_2_ emissions and EE of AAMs. This research contributes significantly towards the implementation and standardization of industrial-scale manufacturing approaches of low carbon footprint AAM mortars in the foreseeable future, particularly in geographic locations with abundance of volcanic ashes, and East Asian countries that have extensive production of fly ash and pal oil fly ash. Furthermore, the final weights and biases of the trained ANN can be used to design AAMs with targeted mechanical properties and CO_2_ emissions based on locally available industrial by products.

## 2. Materials and Methods

### 2.1. Raw Materials Properties

In this research, pure GBFS obtained from an industry supplier in Ipoh, Malaysia, was used as received without any further treatment and utilized as a main resource of calcium materials in AAM production. The low calcium fly ash (FA) was collected from the Tanjung Bin power station in Johor, Malaysia, and used as received. Raw POFA was collected from the local palm oil industry in Malaysia. Incomplete combusted fibres and kernel shells were separated using a 300 µm sieve before drying in an oven for 24 h at 105 ± 5 °C to remove moisture. The POFA was ground using a Los Angeles machine to obtain an average particle size of 10 µm. To achieve the desired level of fineness, the POFA was crushed for 12,600 cycles over 6 h. Moreover, homogeneous tile ceramic waste was collected from the White Horse ceramic manufacturer in Pasir Gudang Johor, Malaysia, which was with no glassy coating. It was crushed using a jaw crusher, then sieved on a 600 μm sieve to remove large size particles. The ceramic waste particles that passed through the 600 μm sieve were ground for 6 h using a Los Angeles abrasion apparatus with 20 stainless steel balls having 40 mm in diameter, thereby obtaining the final waste ceramic powder (WCP). FA, POFA and WCP were used as source aluminosilicate materials for making AAMs. The colours of the GBFS, FA, POFA, and WCP were off-white, light grey, dark grey and light grey, respectively. In terms of physical properties, the lower specific gravity was observed for POFA (1.96) compared to 2.2, 2.6, and 2.9 for FA, WCP and GBFS, respectively. The average particle size of GBFS, FA, POFA and WCP was 12.8, 10.0, 8.2 and 35 μm, respectively.

### 2.2. Design of Ternary Blended Alkali-Activated Materials (AAMs)

Using X-ray fluorescence spectroscopy (XRF, HORIBA, Singapore, Singapore), the chemical compositions of the industrial byproduct materials were determined, as given in Table 1. It was revealed that the main compound in POFA, FA, and WCP, was SiO_2_ (64.2%, 57.2%, and 72.6%, respectively), whereas in GBFS, CaO was the main compound (51.8%). Al_2_O_3_, SiO_2_, and CaO are essential oxides throughout the hydration and production processes of the C-(A)-S-H gels. Nevertheless, the low contents of Al_2_O_3_ and CaO in WCP require adding materials comprising high quantities of Al_2_O_3_, such as FA, and CaO rich materials, such as GBFS, to produce high-performance alkali-activated binders. According to ASTM C618-15 [26], FA and WCP are classified as class F pozzolans due to the existence (higher than 70%) of SiO_2_ + Al_2_O_3_ + Fe_2_O_3_.

**Figure 1:**
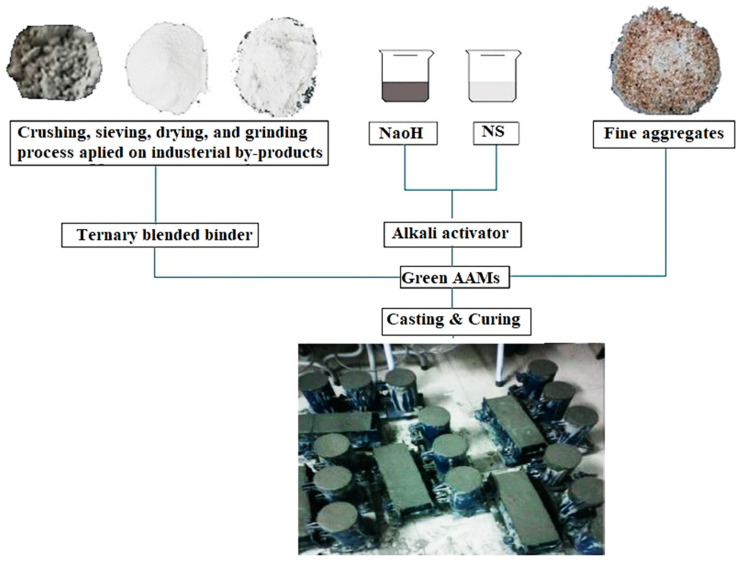
Production stage of alkali-activated materials (AAMs).

Figure 1 depicts the production stage of alkali-activated green mortar, using ternary blended industrial byproducts. Ternary blended AAMs were examined to determine the influence of calcium oxide on the geopolymerization process. Using trial mixtures, the optimal ratio of sodium silicate-to-sodium hydroxide alkali activators, sodium hydroxide molarity, binder-to-aggregate ratio, and alkaline solution-to-binder ratio were determined as 0.75, 4M, 1, and 0.4, respectively, hence these values were fixed for all AAMs. Analytical grade sodium silicate solution “Na_2_SiO_3_” (NS), comprised of SiO_2_ (29.5 wt %), Na_2_O (14.70 wt %), and H_2_O (55.80 wt %), in combination with sodium hydroxide (NaOH) were used as the alkali activator to prepare the proposed AAM mixtures. NaOH pellets were dissolved in water to make the alkaline solution with 4M concentration. In the first phase, the solution was cooled for 24 h and then added to the sodium silicate (NS) solution to obtain an alkaline activator solution with a modulus ratio (SiO_2_ to Na_2_O) of 1.02. The ratio of NS to NaOH was fixed to 0.75 for all the alkaline mixtures.

### 2.3. Testing Procedures

After 365 days of curing at lab temperature of 27 ± 1.5 °C and relative humidity of 75%, the compressive strength test was carried out as per ASTM C109-109M [27] guidelines. Three samples from each mixture were tested at this age and the average value is reported. Upon sample preparation, each test specimen was centred precisely between the top and bottom metal bearing platens of a hydraulic press machine. A consistent loading rate of 2.5 kN/s was applied to the samples. Density and compressive strength figures, based on the weight and size of the test samples, were automatically generated owing to the test machine’s capabilities.

Ultrasonic pulse velocity (UPV) testing can be deployed in-situ as a non-destructive evaluation technique to check the quality of concrete in terms of material discontinuities and damage such as cracks and delaminations under a given exposure time. In this test, the strength and quality of concrete are appraised by measuring the velocity of an ultrasonic pulse passing through the concrete element. The pulse velocity can be determined by measuring the length between the transducers and the travel time, as per Equation (1) where, *x* is distance and *t*, is the transit time. More rapid velocity indicates better material integrity, higher density, and superior quality of the material.

(1) $$\mathrm{UPV}=v_{c}\left(x,\;t\right)=x/t$$

### 2.4. Life-Cycle Assessment (LCA) Approach in AAMs

The LCA of any product, activity or service, is typically inventory-based, in which raw materials, energy, and environmental emissions are identified [28]. The LCA analysis provides a massive multi-dimensional set of input and output data that are difficult to interpret and comprehend. Additional precautions are generally recommended while relating input to output data in the LCA product system to avoid uncertainties in impact analysis [29].

In this research, the main objective of LCA was to contrast the production of AAMs manufactured with ternary blended industrial by products, with a benchmark conventional OPC-based mortar. The LCA was concerned with CO_2_ emissions and EE in compliance with the Inventory of Carbon and Energy (ICE) [30] in which the system boundary begins with the raw material acquisition (cradle) and ends at the factory gate, exclusive of the impacts associated with transportation, service or use life, and end-of-life. Table 2 shows the CO_2_ emissions and EE for all the binder materials, OPC, and fine aggregate used as provided by ICE.

**Figure 2:**
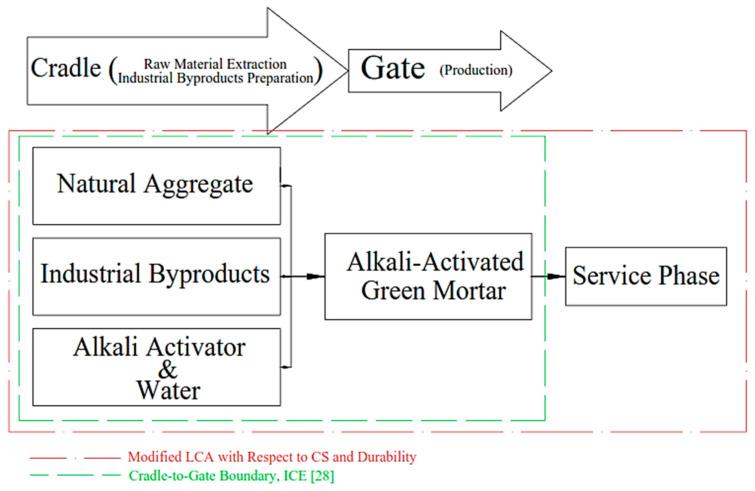
Cradle-to-gate and modified LCA boundary.

The LCA technique considers different scenarios and possibilities to minimize raw material and energy consumption and decrease the environmental footprint of construction materials. The processes and assumptions considered in these scenarios should be based on current technological developments and standard practices. This approach may be conservative, particularly for recycling and reuse of materials, for which major improvements are expected, considering that the market for secondary materials is still at an early stage of development [31,32]. The first scenario for LCA in this research is cradle-to-gate, which is characterized by the major processes associated with raw materials extraction and materials production stages, in compliance with ICE [30,33,34,35]. Figure 2 depicts the cradle-to-gate of ternary blended AAMs. Such a conventional scenario was already applied to “green” concrete containing industrial byproducts, AMMs and geopolymers made with FA, GBFS, MK, along with alkali activators such as sodium hydroxide [36,37,38]. Nevertheless, such a traditional scenario is not sufficiently reliable for assessing the environmental impacts (or benefits) of green concrete products since it precludes the advantageous effects of alkali-activated binders composed of industrial byproducts on the mechanical properties and durability. Only a dearth of research considered the normalization of the climate change potential with respect to green concrete’s mechanical properties [39]. Since the life span of concrete and mortar can be extended via improving the durability and mechanical properties, these parameters should be captured in the domain of the LCA criteria. Thus, in the present research, by defining another scenario, the cradle-to-gate LCA is adapted by taking the mechanical properties and durability of the AAMs into consideration. Using this approach, not only the impact of material manufacturing is accounted for, but also the impacts of service life are incorporated in the LCA criteria.

The functional unit of CO_2_ emissions and EE is per cubic meter of AAM. Additionally, a revised cradle-to-gate system boundary was applied to the AAMs to include the service life phase on the basis of performance criteria. Accordingly, the service life impacts were incorporated through consideration of the CS and durability (sulfuric acid and sulphate resistance) of AAMs. The following equation was considered to estimate the CO_2_ emission and EE per cubic meter of the AAMs:

(2) $$Total\;{CO}_{2}\;emission\;or\;EE=\overset{n}{\underset{i=1}{\sum }}mi\left(pi\right)$$

where the left-hand side of the equation indicates the net amount of CO_2_ emission (kg CO_2_) and EE (MJ) for every cubic meter of AAM production, mi indicates the fraction of component *i*, and pi specifies the CO_2_ emissions (kg) and EE (MJ) per cubic meter of component *i* produced.

## 3. Results and Discussion

### 3.1. Mechanical and Durability Performance of AAMs

Four ternary blended AAMs were investigated, where at each level, the GBFS percentage, as a source of CaO, remained constant at a minimum of 20% in the replacement process and a maximum of 70%. Table 3 reports the compressive strength (CS) for all 42 AAM mixtures. It can be observed that highest mechanical properties were achieved by AAMs made with a high volume of GBFS, while AAMs made with a high volume of POFA resulted in the lowest mechanical properties. The mechanical properties of AAMs made with a high volume of WCP also were not satisfactory. However, increasing the GBFS dosage in the binder mass improved the mechanical strength in this category. The mechanical features in AAMs made with a high-volume FA were significantly dependent on the percentage of GBFS in the binder mass, where substituting GBFS by POFA significantly decreased the CS.

Considering the observed results, it can be argued that the CS of the AAM mixtures significantly depended on the percentage of GBFS as acknowledged by previous literature. For instance, it was proposed by [40] that the proper GBFS content in an alkali-activated fly ash/slag mixture was determined to be 15–20% of the total binder by weight considering the setting time and compressive strength of the alkali-activated fly ash/slag concrete cured at room temperature. Other researchers [41] investigated the mechanical properties and hydration of alkali-activated blended Portland cement produced from a mixture of 80% granulated blast furnace slag and 20% Portland. They concluded that the hybrid cement achieved compressive strength of 4.5 and 10.8 times higher than the reference (100% Portland cement) when activated by NaOH and waterglass (sodium silicate + NaOH) respectively. Overall, Table 3 reveals that the average CS of the studied AAMs mixtures was 61.3 MPa, which is satisfactory, while having much lower embodied energy and CO_2_ emission compared to benchmark traditional OPC-based mortars.

Generally, sulfuric acid can attack AAMs by dissolving the binder paste matrix, thus weakening the mechanical properties of the AAM mortar. In this research, using deionized water, a 10% H_2_SO_4_ acid solution was prepared, and its effects on the residual CS, mass loss, and UPV of AAMs were investigated at the age of 28 and 365 days, in compliance with ASTM C267 specifications [42]. To sustain the pH of the solution over the span of the test duration, the solution was changed every two months. Sulphate attack on the studied alkali-activated mortar specimens was caused by the sulphate ions (SO_4_)^2−^ that were transmitted into the mortar from varying concentrations in the water together with magnesium, calcium, or sodium cations. Magnesium sulphate solution was also employed to evaluate the resistance to sulfate attack of the alkali-activated specimens using a test procedure similar to that adopted for the sulfuric acid attack test.

**Figure 3:**
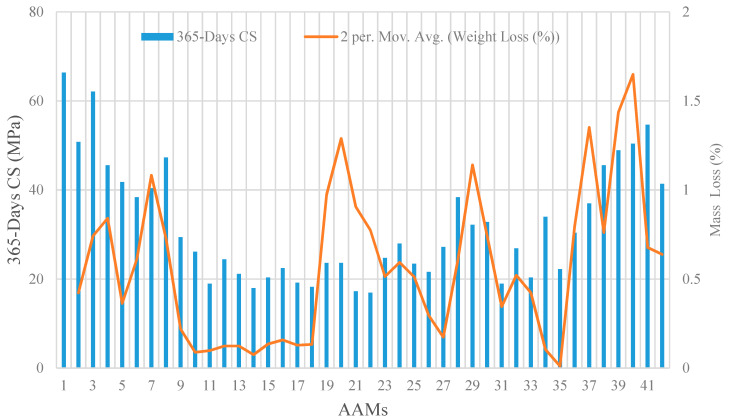
Effects of exposure to sulfuric acid solution on CS and mass loss of AAM mortars.

**Figure 4:**
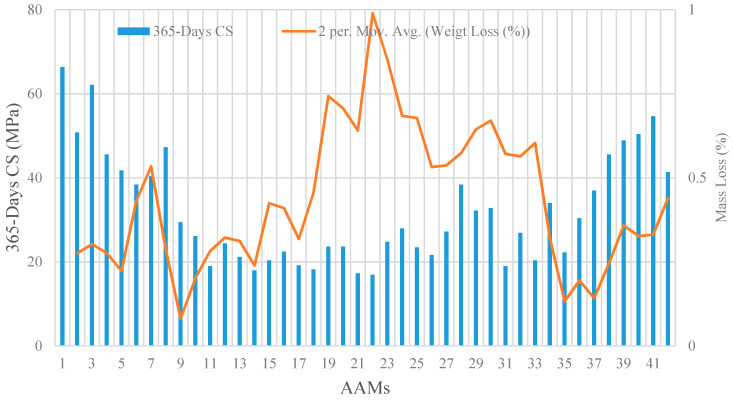
Effects of exposure to sulphate solution on CS and weight of AAM mortars.

Figure 3 illustrates the residual CS and mass loss of all the 42 AAM mixtures after 365-days of immersion in the sulfuric acid solution. On average, the CS and specimen mass declined by 90 and 0.56 percent, respectively, compared to the control intact specimens. The maximum reduction in CS was inflicted to specimens in the category of high-volume GBFS by around 300%, while the specimens with high-volume WCP experienced major mass loss of an average 0.85%. Figure 4 illustrates the residual CS and mass loss of all 42 AAMs after 365-days of immersion in the sulphate solution. There was generally a similar pattern observed for residual CS compared to that of immersion in the sulfuric acid solution, whereas the maximum mass loss was recorded for specimens with high-volume GBFS by an average of 0.66%.

**Figure 5:**
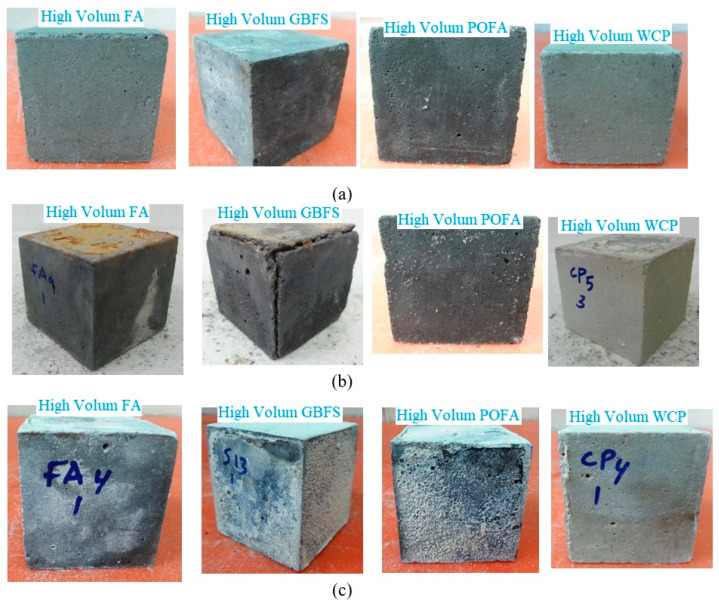
Physical appearance of cubic AAM mortar specimens made with different industrial byproducts: (**a**) control sample, (**b**) after 365-days of immersion in sulfuric acid solution, and (**c**) after 365-days of immersion in sulphate solution.

Figure 5 displays the physical appearance of the cubic AAM mortar specimens prepared with different industrial byproducts after 365-days of immersion in the sulfuric acid and sulphate solutions. Comparing Figure 5b (after 365-days of immersion in the sulfuric acid solution) to the control intact samples (Figure 5a), it can be observed that the durability of AAM mortar specimens exposed to the sulfuric acid environment gradually decreased with increasing GBFS content. However, increasing the level of FA, POFA and WCP from 30% to 70% led to increased resistance of AAM mortar specimens to sulfuric acid attack, indicating excellent durability performance. Upon exposure of the AAM mortar specimens to the sulfuric acid solution, the Ca(OH)_2_ compound in mortar reacted with SO_4_^−2^ ions and formed gypsum (CaSO_4_·2H_2_O). This caused expansion in the alkali-activated matrix and additional cracking in the interior of specimens, as per the visual appearance of these specimens. The high calcium oxide in the high-volume GBFS AAM specimens compared to that in the other matrixes resulted in more abundant gypsum formation.

Therefore, degradation in residual CS along with more substantial mass loss were observed for AAM mortar specimens made with high-volume GBFS and immersed in the sulfuric acid solution. A reduction in mass loss can be explained by increasing the SiO_2_ and Al_2_O_3_ contents associated with a reduction of the CaO content. Moreover, decreasing the CaO content reduced gypsum formation, thus increasing the durability of the alkali-activated mortar specimens.

For the durability to the sulphate environment, it was found that increasing the FA, POFA, and WCP levels in the alkali-activated matrix mitigated the deterioration (Figure 5c) and increased the residual strength. Several researchers have reported that sulfate deterioration can cause mechanical strength loss, expansion, spalling of surface layers, and ultimately disintegration. Most experts attribute sulphate attack to the formation of expansive ettringite (3CaO·Al_2_O_3_·3CaSO_4_·32H_2_O) and gypsum (CaSO_4_·2H_2_O), which may be accompanied by expansion or softening.

**Figure 6:**
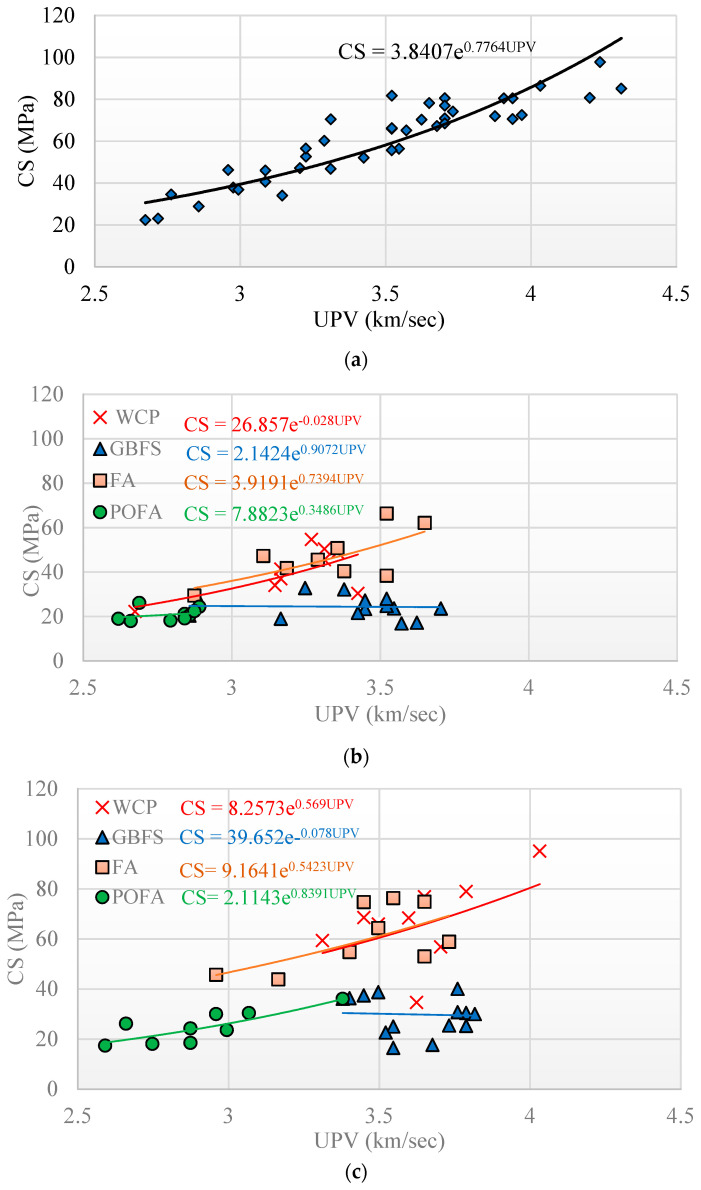
Relationship between ultrasonic pulse velocity (UPV) and CS for all 42 AAM mixtures: (**a**) original condition, (**b**) after immersion in sulfuric acid solution, and (**c**) after immersion in sulphate solution.

The experimental results confirm that pulse velocity decreased by an average of about 8% and 5% for specimens immersed in acid and sulphate solutions for a period of 365 days, respectively. In this study, using non-linear regression analysis, an exponential function for estimating the relationship between CS and pulse velocity of AAMs was established. Figure 6 depicts the relationship between the mean values of UPV and CS for all the 42 AAM mixtures investigated before and after 365 days of immersion in the sulfuric acid and sulphate solutions. The results confirm that there was an inverse correlation between CS and pulse velocity reduction, where AAMs with lower CS have shown larger reduction of pulse velocity. The highest pulse velocity before and after immersion in the sulfuric acid and sulphate solutions was achieved by AAM mixture 20 made with 20% FA + 70% GBFS + 10% POFA, with a CS of 97.75 MPa. Generally, AAMs with higher dosage of GBFS exhibited the highest value of pulse velocity before and after immersion in the sulfuric acid sulphate solution compared to other mixtures. However, AAMs incorporating high-volume WCP demonstrated appropriate performance in resisting exposure to the sulphate solution, where the average pulse velocity and CS remained nearly unchanged before and after the exposure. This can be explained by the morphology of this alkali-activated mixture which possess high magnesium sulphate (MgSO_4_) and silicon dioxide SiO_2_ contents, providing resistance against sulphate attack. Previous literature indicated the relationship between CS and pulse velocity as a measure of material deterioration, internal cracking, and pre-existing defects in mortars before and after immersion in sulfuric acid and sulphate solutions using the following exponential function [43,44,45], where *V* is the UPV, and the coefficients *A* and *B* are empirical constants.

(3) $$CS=Ae^{\left(BV\right)}$$

### 3.2. Cradle-to-Gate LCA

**Figure 7:**
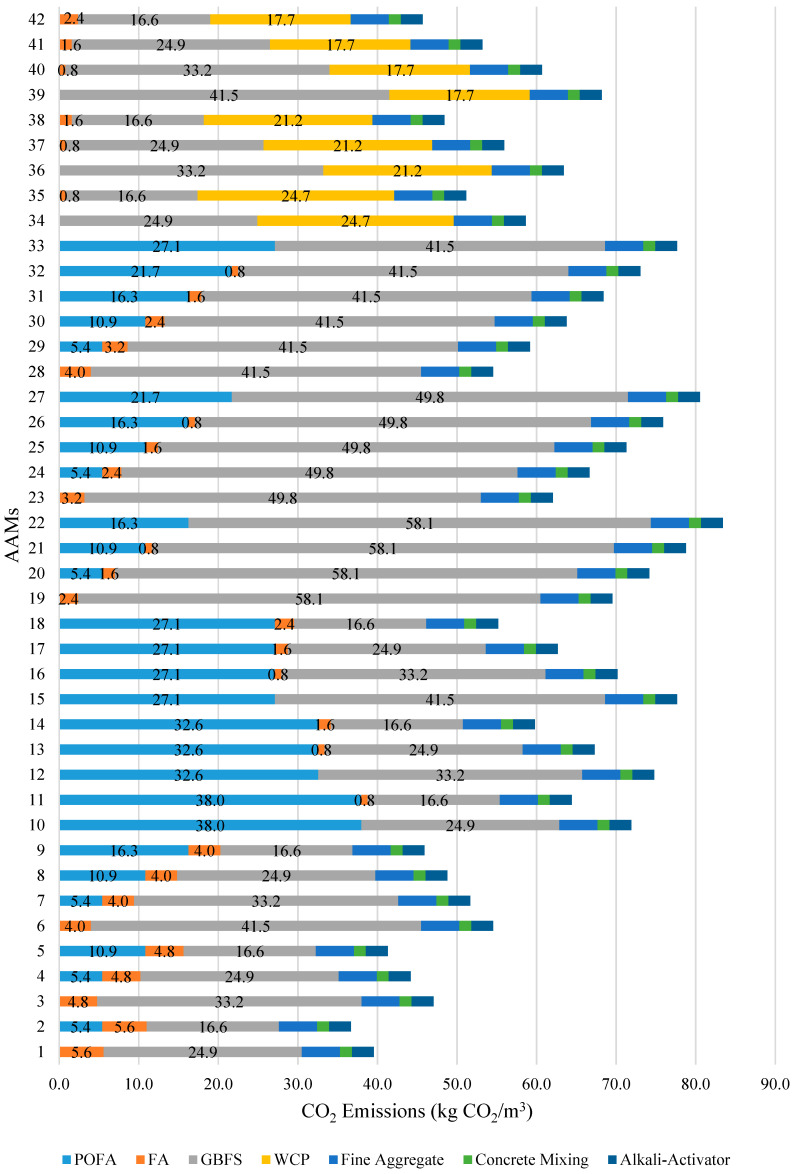
Distribution of CO_2_ emission by AAMs ingredient and phase.

**Figure 8:**
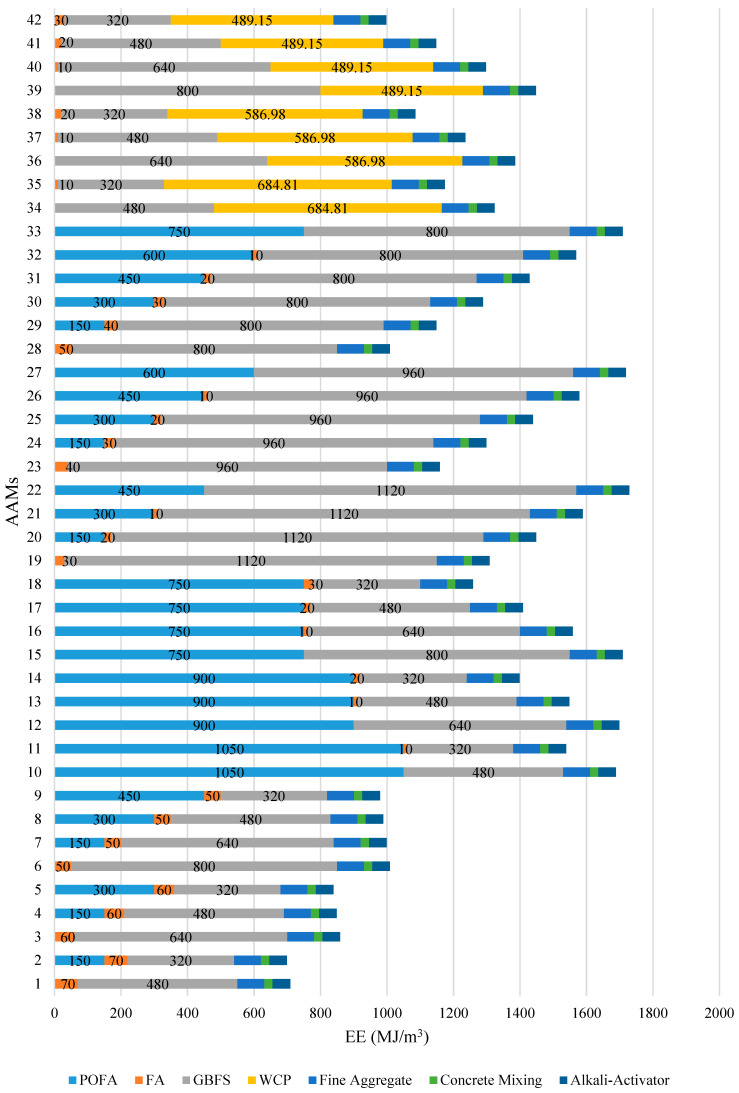
Distribution of EE by AAMs ingredient and phase.

The estimated CO_2_ emissions and EE per cubic meter of AAM for all the 42 mixtures explored are illustrated in Figure 7 and Figure 8, respectively. The percentage distribution of CO_2_ emissions and EE associated with the production of non-cementitious materials, fine aggregate, mixing, and alkali activator, were considered constant for all the AAM mixtures. The results indicate that the AAM mixture with high-volume FA emitted the least amount of CO_2_ and consumed the least amount of energy with an average of 45.5 kg CO_2_/m^3^ and 881.2 MJ/m^3^, respectively. On the other hand, the AAM mixture made with high-volume GBFS emitted the highest CO_2_ amount, while the AAM mixture made with high-volume POFA consumed the highest amount of energy with an average of 70.6 kg CO_2_/m^3^ and 1534.5 MJ/m^3^, respectively. The results confirm that the CO_2_ emissions and energy consumption associated with the production of AAM made with GBFS and POFA are relatively higher compared to that of AAM made with other industrial waste materials. Such results can be explained by the higher amount of electricity required for grinding GBFS to obtain the recommended particle size and for drying POFA in the oven at a temperature of 110 ± 5 °C for 24 h. Overall, it can be concluded that the highest CO_2_ emissions and EE of all 42 AAM mixtures studied were significantly lower than that of the benchmark conventional mortar prepared using OPC (1/3 cement-sand mix), which is associated with 436.8 kg CO_2_/m^3^ and 2793 MJ/m^3^, respectively.

### 3.3. Modified LCA with Respect to Compressive Strength (CS) and Durability

**Figure 9:**
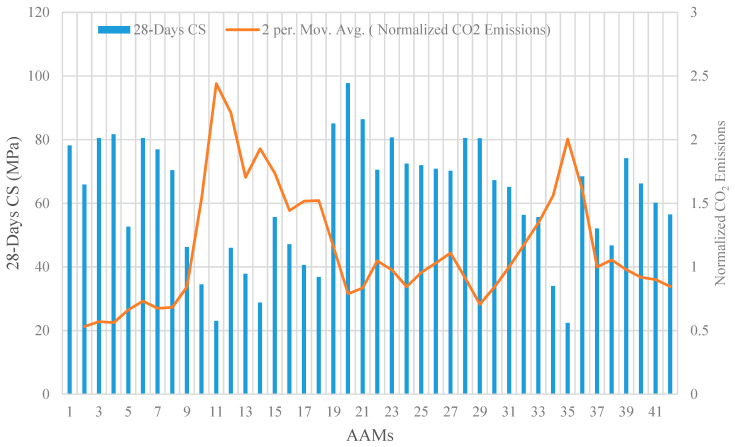
Illustration of compressive strength versus normalized CO_2_ emissions.

For a consistent and systematic comparison among AAM mixtures, their CO_2_ emissions are normalized with respect to CS, as shown in Figure 9. The results confirm that in AAM mixtures incorporating high-volume FA and GBFS, a lower intensity of normalized CO_2_ emissions is achieved. The highest intensity of normalized CO_2_ emissions was recorded for AAM mixtures containing high-volume POFA, which is correlated to its relatively low CS and high CO_2_ emission. For a given CS, a lower intensity of normalized CO_2_ emissions can be achieved by replacing FA with GBFS. For instance, at CS of around 80 MPa, a reduction in GBFS from 70% (Mixture 9) to 30% (Mixture 1) reduced the normalized CO_2_ emission from 1.15 CO_2_·m^−3^/MPa to 0.5 CO_2_·m^−3^/MPa. By substituting 20% of WCP mass (Mixture 35) with FA (Mixture 41) in AAMs containing high-volume WCP, the intensity of normalized CO_2_ emissions could be decreased by around two times.

**Figure 10:**
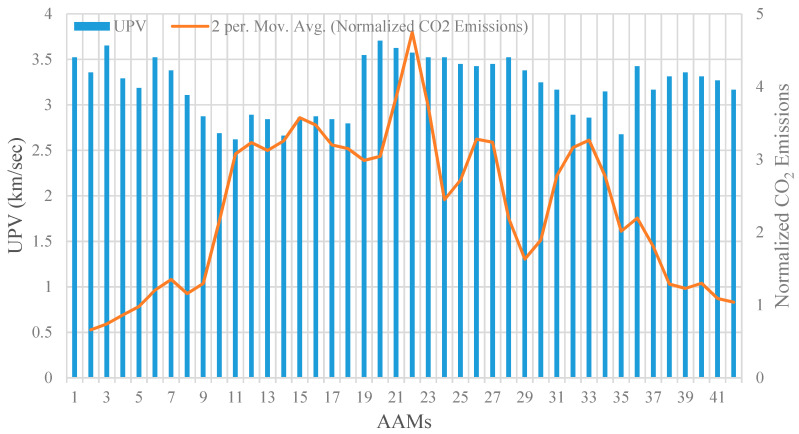
UPV versus normalized CO_2_ emission subjected to sulfuric acid attack.

**Figure 11:**
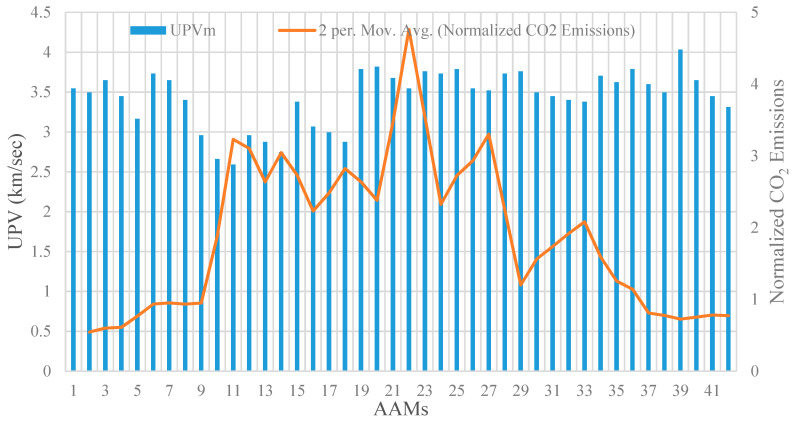
UPV versus normalized CO_2_ emission subjected to sulphate attack. Add unit for UPV.

To include durability in the performance criteria of the studied AAM mixtures, their CO_2_ emissions were normalized with respect to CS after 365 days of immersion in the sulfuric acid and sulphate solutions, as shown in Figure 10 and Figure 11. The results confirm that the normalized CO_2_ emissions for AAM mixtures made with high-volume POFA and GBFS were relatively higher than that for other mixture designs. This can be ascribed to the fact that the mixtures containing GBFS and POFA were vulnerable to sulfuric acid and sulphate attack, where their CS significantly decreased after 365 days of immersion in these solutions. The highest normalized CO_2_ emission in both sulfuric acid and sulphate attack was achieved by the AAM mixture number 22 incorporating high GBFS and POFA, with an intensity of around 5 CO_2_·m^−3^/MPa, which is nearly 5 times higher compared to that of the intact control condition. The intensity of normalized CO_2_ emissions in AAMs made with a high percentage of GBFS and POFA did not experience major changes compared to their original intact conditions, where the average intensity in the sulfuric acid and sulphate attack exposures were around 1.26 and 0.83 CO_2_·m^−3^/MPa, respectively.

## 4. Artificial Neural Network (ANN) for Estimating CO_2_ Emission and Embodied Energy (EE)

### 4.1. Modeling Methodology

An ANN combined with a metaheuristic algorithm was developed to estimate CO_2_ emissions and EE of AAM mixtures. The model’s final weight and bias values can be used to design AAM mixtures with targeted CO_2_ emissions and energy consumption based on available local waste materials. The multilayer feed-forward network provides a reliable feature for ANN structures and was thus used in this research. This network comprises three individual layers: the input layer, where the data are defined to the model; the hidden layer/s, where the input data are processed; and finally, the output layer, where the results of the feed-forward ANN are produced. Each layer contains a group of nodes referred to as neurons that are connected to the proceeding layer. The neurons in the hidden and output layers consist of three components: weights, biases, and an activation function that can be continuous, linear, or nonlinear. Standard activation functions include non-linear sigmoid functions (logsig, tansig) and linear functions (poslin, purelin) [46]. Once the architecture of a feed-forward ANN (number of layers, number of neurons in each layer, activation function for each layer) is selected, the weight and bias levels should be adjusted using training algorithms. One of the most reliable ANN training algorithms is the backpropagation (BP) algorithm, which distributes the network error to arrive at the best fit or minimum error [47,48] and was, accordingly, used in this study.

#### 4.1.1. Cuckoo Optimization Algorithm (COA)

Bird species lay eggs for reproduction. Finding a safe nest to lay and hatch their eggs and raise the chicks to the point of independence is always a challenge for birds. Therefore, birds use different approaches, including intricate design, artistry, and complex engineering so that even all-seeing eyes can hardly find them. Other birds ignore every conventional form of parenthood and homemaking and rely on a gimmick to raise the young. These categories of birds, the so-called “brood parasites,” lay their eggs in the nest of other species instead of building their own nests, leaving those parents to take care of their chicks. A well-known brood parasite is the cuckoo, which is skillful in the art of cruel deception [49]. The cuckoo starts with an initial population. They make some eggs that they manage to lay in the nest of several other host birds. This strategy involves speed, stealth, and surprise, where the mother takes away one egg laid by the host and lays her own egg. They carefully imitate the pattern and color of their own eggs to match that of their hosts. Some of these eggs, which are more similar to the host bird’s eggs, will have a better chance of growing and becoming an adult cuckoo. Other eggs are detected and destroyed by the host bird. The number of eggs grown indicates the suitability of the nests in that area. The more eggs that can survive in an area, the more profit (desire) will be allocated. Therefore, the situation in which the largest number of eggs is saved will be a parameter that they intend to optimize [49]. The cuckoo optimization algorithm (COA) is based on the above logic and is coupled here with the ANN model.

#### 4.1.2. Generation of Training and Testing Data Sets

**Figure 12:**
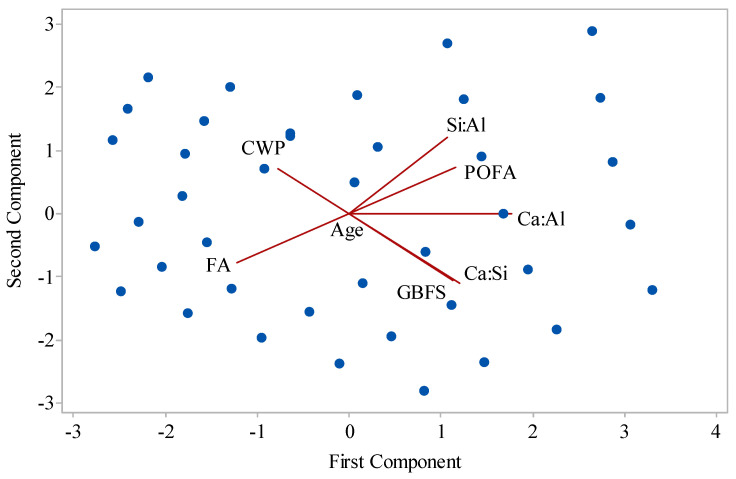
Scatter graph of the total density of input parameters using principal component analysis (PCA).

To train and develop a reliable ANN, the chemical properties of the industrial byproducts, see Table 2, were taken into account on the basis of input variables. The input and output variables along with their properties are given in Table 4. It can be observed in this table that the number of input and output variables are 8 and 2, respectively. Since a large number of input parameters in ANN generally tend to increase the error, principal component analysis (PCA) was considered to make the input parameters orthogonal to each other. Accordingly, the input density diagram is shown in Figure 12.

PCA is a dimension-reduction tool that can be used to reduce a large set of variables to a small set that still contains most of the information in the original large set. This can be achieved by applying a transformation function, the so-called principal components (PC), on the primary variables. PCs are unrelated to each other and are sorted in such a way that the primary variables contain the most features of variance of the primary variables. The detailed information of this method can be found in [50,51]. Using PCA, Table 5 reports the influence of each parameter on inputs variables. It can be observed in this table that the conversion of 8 input parameters into four variables, PCA 1 to PCA 4, resulted in using 98.8% of the data and, as a consequence of such data convergence, better model results can be obtained. The resulting input variables using PCA are shown in Table 6.

Therefore, according to the optimal accuracy of the PCA method, four input variables were used in the ANN model. The number of hidden layers and total number of neurons in the hidden layers in an ANN depends on the nature of the problem [52]. Generally, a trial and error method is used to obtain a suitable architecture that best reflects the characteristics of the laboratory data. In the present study, an innovative method for calculating the number of neurons in the hidden layers was considered, as shown in the equation below, where *NH* is the number of neurons in the hidden layers and *NI* is the number of input variables [53].

(4) $$N_{H}\leq 2N_{I}+$$

Since the number of effective input variables is 4, the empirical equation shows that the number of neurons in hidden layers can be less than 9. Therefore, several networks with different topologies, with a maximum of two hidden layers and a maximum of 9 neurons, were trained and explored in this study. The hyperbolic tangent stimulation function and Levenberg–Marquardt training algorithm were used in all networks. The statistical indices used to evaluate the performance of different topologies are the root mean squared error (*RMSE*), average absolute error (*AAE*), model efficiency (*EF*), and variance account factor (*VAF*), which are defined as follows [54]:

(5) $$RMSE={\left[\frac{1}{n}\overset{n}{\underset{i=1}{\sum }}{\left(P_{i}-O_{i}\right)}^{2}\right]}^{\frac{1}{2}}$$

(6) $$AAE=\frac{\left|\sum_{i=1}^{n}\frac{\left(O_{i}-P_{i}\right)}{O_{i}}\right|}{n}\times 100$$

(7) $$EF=1-\frac{\sum_{i=1}^{n}{\left(P_{i}-O_{i}\right)}^{2}}{\sum_{i=1}^{n}{\left({\bar{O}}_{i}-O_{i}\right)}^{2}}$$

(8) $$VAF=\left[1-\frac{var\left(O_{i}-P_{i}\right)}{var\left(O_{i}\right)}\right]\times 100$$

**Figure 13:**
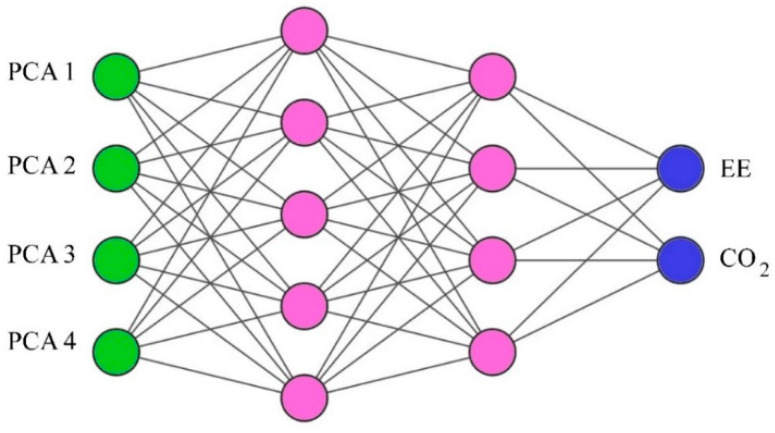
Topology of a feed-forward artificial neural network (ANN) with two hidden layers (4–5–4–2 structure).

After examining different ANN model topologies, it was found that the network with a 4–5–4–2 topology had the lowest value of error in *RMSE*, *AAE*, *EF*, *VAF* and the highest value of *R*^2^ to estimate the two output parameters. It should be emphasized that the error criteria for training and testing the data are calculated in the main range of variables and not in the normal range. Figure 13 illustrates the topology of a feed-forward ANN network modified by PCA with two hidden layers, four input variables (neurons), and two output parameters.

**Figure 14:**
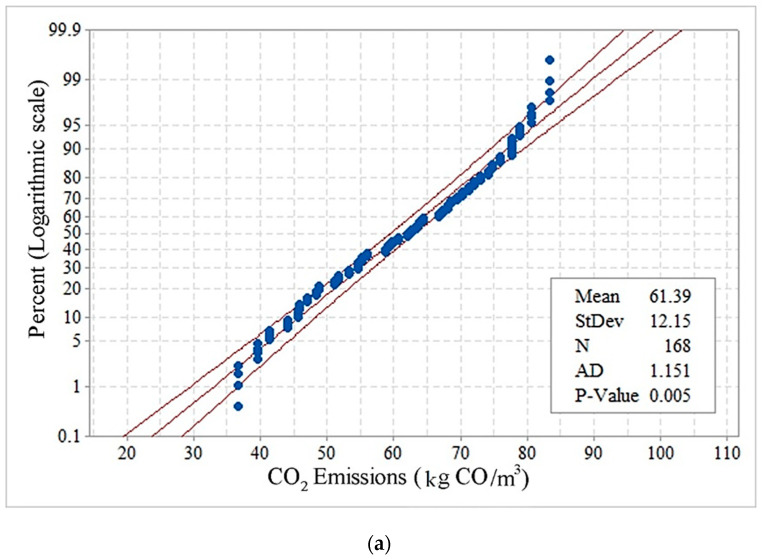

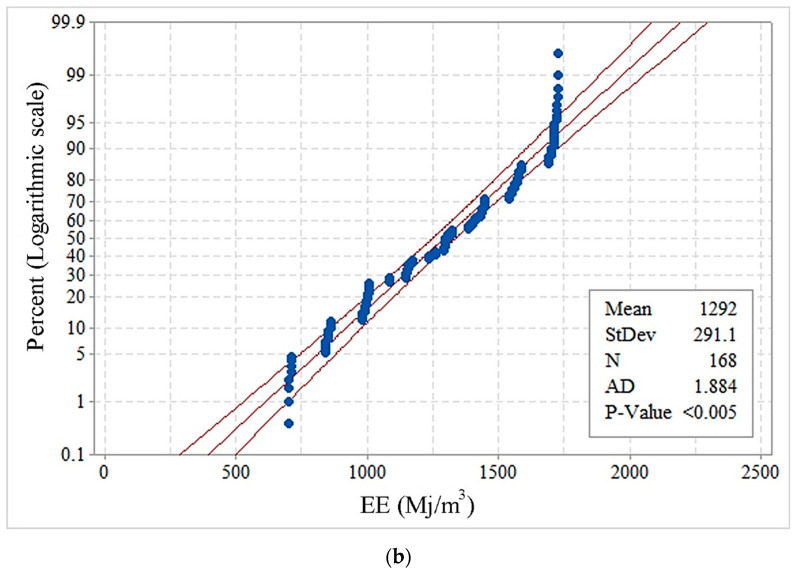
Probability plot diagrams for (**a**) CO_2_ emissions, and (**b**) EE.

The ANN used in this study was the Newff Feed Forward; 70% of the experimental data (118 data units), out of 168 experimental data, was used for training, and the remainder 30% (50 data units) was used for network testing. To optimize the ANN’s weights and biases, the COA was used to provide the least prediction error for the trained structure (modified with PCA). The properties of the COA parameters are shown in Table 7. Also, considering that the statistical behavior of the output data (EE and CO_2_) should be evaluated, probability plot diagrams related to determining their normal distribution were examined. The results showed that their statistical behavior followed a normal distribution, as illustrated in Figure 14.

### 4.2. Model Predictions and Results

**Figure 15:**
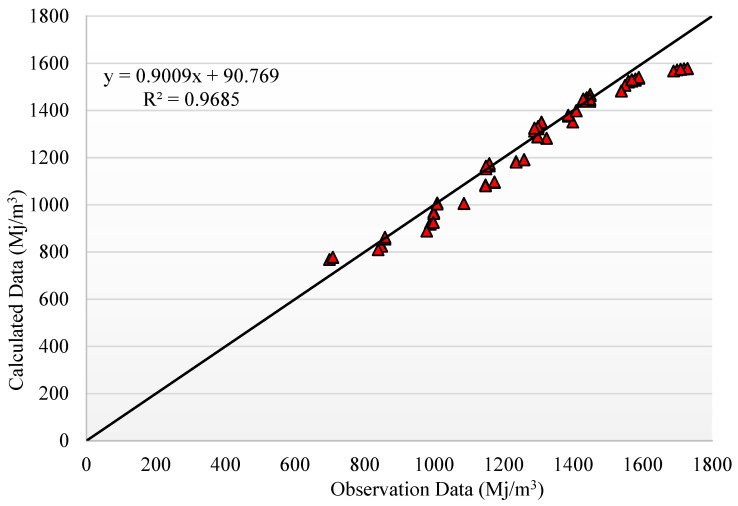
Predicted vs. experimental values of EE estimated by the PCA-COA-ANN model.

**Figure 16:**
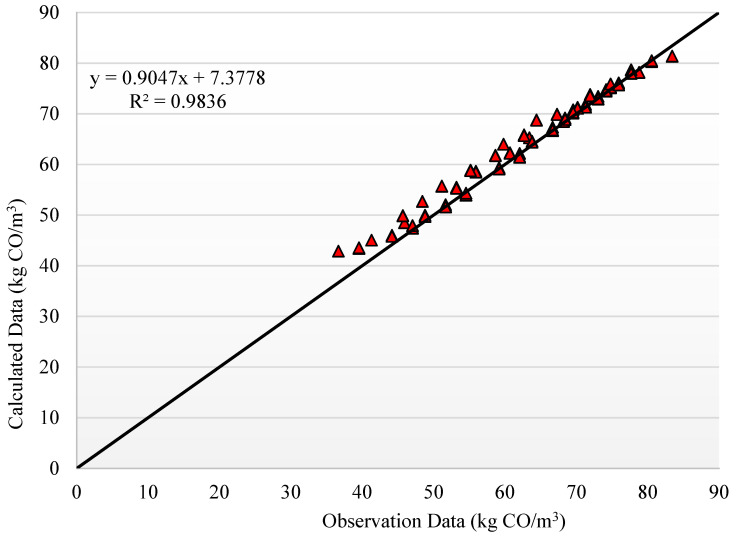
Predicted vs. experimental values of CO_2_ emissions estimated by the PCA-COA-ANN model.

The results of the trained and optimized PCA-COA-ANN model are depicted in Figure 15 and Figure 16 for the EE and CO_2_ emissions output parameters, respectively. The results indicate that the PCA-COA-ANN estimated reliable and accurate values for the ratio of observational to computational values, *R*^2^, for both input parameters, indicating high accuracy and robustness of the proposed model. Table 8 provides the final weights and biases for both hidden layers estimated by the PCA-COA-ANN model. Using the values of these weights and biases between the different ANN layers, the two output parameters (EE and CO_2_ emissions) can be determined and predicted. Moreover, these final weight and bias values can be used to design AAMs with targeted mechanical properties and CO_2_ emissions with respect to the availability of industrial byproducts and environmental constraints. Accordingly, rather than executing extensive and laborious experimental programs to reach reasonable results, the trained model could be run in a very short time to obtain near optimal results. Only limited experimental validation could be carried out thereafter to ensure that variability in local materials and experimental equipment and procedures do not alter the model predictions significantly. Moreover, the experimental validation data could be cumulated and used further in model training and fine tuning for local conditions, which could save time and cost in AAM mixture design development.

### 4.3. Sensitivity Analysis

Sensitivity analysis (SA) reveals how significantly the model’s output is affected by changes within input variables. There are two main types of SA: global and local sensitivity analysis, where the local sensitivity analysis concentrates on the local impact of individual input parameters on the overall performance.

Conversely, the global sensitivity analysis (GSA) evaluates the influence of individual input parameters over their entire spatial range and measures the uncertainty of the overall performance (output) caused by input uncertainty, over the interaction with other parameters, or taken individually. Therefore, considering the nature of the EE and CO_2_ emission parameters in this study, GSA is more rational for assessing the impact of input parameters on the overall performance.

Amongst diverse GSA methods, a variance-based approach was primarily considered in the previous literature for sensitivity analysis [55]. The method provides a specific methodology for defining the total and first-order sensitivity indices for each input parameter of the ANN model. Assuming a model of the form *Y* = *f* (*X*_1_, *X*_2_,…, *X_k_*), where *Y* is a scalar, the variance-based technique takes a variance ratio to evaluate the impact of individual parameters using variance decomposition as per the following equation:

(9) $$V=\sum_{i=1}^{k}V_{i}+\sum_{i=1}^{k}\sum_{j>i}^{k}V_{ij}+\ldots +V_{1,2,\ldots ,k}$$

where *V* is the variance of the ANN model output, *Vi* is the first-order variance for the input *X*, and *Vij* to *V*_1,2, …, *k*_ corresponds to the variance of the interaction of the *k* parameters. *Vi* and *Vij*, which denote the significance of the individual input to the variance of the output, are a function of the conditional anticipation variance, following the equation.

(10) $$V_{i}=V_{x_{i}}\left[E_{x_{\sim i}}(YX_{i}\right]$$

(11) $$V_{ij}=V_{x_{i}x_{j}}\left[E_{x_{\sim ij}}(YX_{i},X_{j}\right]-V_{i}-V_{j}$$

where 𝑋∼𝑖 designates the set of all input variables apart from *Xi*. The first-order sensitivity index (*Si*) represents the first-order impact of an input *Xi* on the overall output provided by the following equation:

(12) $$S_{i}=\frac{V_{i}}{V\left(Y\right)}$$

With this definition, all sensitivity indices can be estimated, where the summation of all $S_{i}$ are equal to one:

(13) $$\overset{n}{\underset{i=1}{\sum }}S_{i}+\underset{i<j}{\sum }S_{ij}+\underset{i<j<k}{\sum }S_{ijk}+\ldots +S_{12\ldots n}=1$$

**Figure 17:**
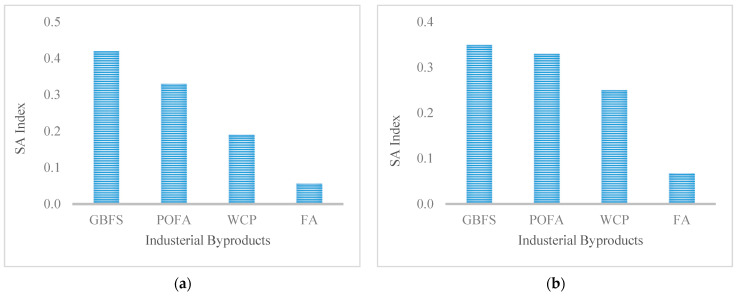
Sensitivity indices of variable (**a**) CO_2_ emissions and (**b**) EE.

The above methodology for calculating the first-order sensitivity index was considered in this research. The results of the sensitivity analysis are presented in Figure 17. It can be observed that, apart from the fine aggregate, mixing, and alkali activator, which were not taken into account in the sensitivity analysis, the results indicate that the percentage of GBFS had a major influence, while the percentage of FA has the least effect on both output parameters of EE and CO_2_ emissions. The POFA can be classified as the second most influential input variable, especially on the EE output parameter.

## 5. Concluding Remarks

This study explored the ‘cradle-to-gate’ LCA of ternary blended alkali-activated mortars composed of industrial byproducts with the system’s boundary extended to include the mechanical and durability properties of AMM mixture designs on the basis of performance criteria. In addition, using the experimental test database thus developed, an optimized ANN model with PCA was combined with the cuckoo optimization algorithm (PCA-COA-ANN) to estimate the CO_2_ emission and embodied energy of AAMs. The following main findings can be drawn from this research:

- The results indicate that the average compressive strength of the studied AAM mixtures was 61.3 MPa, which compares well with traditional cement-based mortars. The highest and lowest mechanical properties were recorded for AAM mixtures made with high contents of GBFS and POFA, respectively.
- On average the residual compressive strength and specimen mass declined by 90% and 0.56%, respectively, after 365 days of immersion in the sulfuric acid solution. The AAM mixtures with high GBFS dosage experienced a major reduction in compressive strength by an average of 300%. In addition, it was found that AAM mixtures made with high WCP and FA contents provided better resistance to both sulfuric acid and sulphate attack.
- Ultrasonic pulse velocity exhibited almost a direct relationship with compressive strength for all AAM mixtures tested. However, after immersion in the sulfuric acid and sulphate solutions, the relationship between the pulse velocity and compressive strength followed an irregular pattern, which depended on the dosage of each industrial byproduct in the mixture.
- The conventional cradle-to-gate LCA revealed that the AAM mixture made with high-volume FA emitted the least amount of CO_2_ and consumed the least amount of energy with average values of 45.5 kg CO_2_/m^3^ and 881.2 MJ/m3, respectively. However, the AAM mixture made with high-volume GBFS and POFA emitted the highest amount of CO_2_ (70.6 kg CO_2_/m^3^) and consumed the highest amount of energy (1534.5 MJ/m^3^), respectively. Nevertheless, these values are significantly lower than that of the benchmark conventional mortar made with pure OPC with 436.8 kg CO_2_/m^3^ CO_2_ emissions and 2793 MJ/m^3^ EE.
- The modified LCA with respect to compressive strength revealed that in AAM mixtures containing high-volume FA and GBFS, lower intensity of normalized CO_2_ emissions was achieved by an average of 0.73 CO_2_·m^−3^/MPa. However, the highest intensity of normalized CO_2_ emissions was achieved by AAM mixtures containing high-volume POFA, with around 1.53 CO_2_·m^−3^/MPa, as correlated to its relatively low compressive strength and high amount of electricity required for oven drying of POFA.
- The modified LCA which included durability in the performance criteria showed that the normalized CO_2_ emission in AAMs containing high-volume POFA and GBFS was relatively higher, with an average intensity of around 3.15 CO_2_·m^−3^/MPa, than that of the other mixture designs. This can be explained by the fact that the mixtures containing GBFS and POFA were vulnerable to sulfuric acid and sulphate attack, where their CS decreased significantly after 365 days of immersion in these solutions.
- For accurate estimation of the output parameters, considering the total number of input variables, principal component analysis was used to reduce the inputs in the ANN. Moreover, the hyperbolic tangent stimulation function and Levenberg–Marquardt training algorithm were used to determine the best topology for the ANN. Several statistical metrics including *RMSE*, *AAE*, *EF*, and *VAF* were used to evaluate the performance of the proposed ANN topology. The PCA-COA-ANN hybrid model provided satisfactory results to estimate the EE and CO_2_ emissions of AAM mixtures, with *R*^2^ values of 0.971 and 0.981 for EE and CO_2_ emissions, respectively.
- Using the optimized weights and biases of the PCA-COA-ANN hybrid model, it is possible to design AAM mixtures with targeted mechanical properties and CO_2_ emissions considering the availability of local industrial by-products.

## Figures and Tables

**Table 1:** Physical and chemical composition of industrial byproduct materials used.

Material	GBFS	FA	POFA	WCP
Specific gravity	2.9	2.2	1.96	2.6
Avr. particle size (µm)	12.8	10	8.2	35
SiO_2_	30.8	57.20	64.20	72.6
Al_2_O_3_	10.9	28.81	4.25	12.6
Fe_2_O_3_	0.64	3.67	3.13	0.56
CaO	51.8	5.16	10.20	0.02
MgO	4.57	1.48	5.90	0.99
K_2_O	0.36	0.94	8.64	0.03
Na_2_O	0.45	0.08	0.10	13.5
SO_3_	0.06	0.10	0.09	0.01
LOI	0.22	0.12	1.73	0.13

**Table 2:** Assumptions used in life-cycle assessment (LCA) calculation (data retrieved from) [30].

Material	CO_2_ Emission (kg CO_2_/kg)	EE (MJ/kg)
POFA	0.0542	1.5
FA	0.008	0.10
GBFS	0.083	1.6
WCP	0.0353	0.9783
Fine Aggregate	0.0048	0.081
OPC	0.73	4.50

**Table 3:** Ternary blended AAM mixtures and calculated EE and CO_2_ emissions.

AAM Designs	Binder Constitution (Composed of Industrial Waste Materials)				Sustainable and Mechanical Features		
	FA	GBFS	WCP	POFA	EE (MJ/m^3^)	CO_2_ Emission (kgCO_2_/m^3^)	28-Days CS (MPa)
High-volume FA mix design							
1	0.70	0.30	0.00	0.00	709.00	39.55	78.18
2	0.70	0.20	0.00	0.10	699.00	36.68	65.89
3	0.60	0.40	0.00	0.00	859.00	47.05	80.51
4	0.60	0.30	0.00	0.10	849.00	44.18	81.70
5	0.60	0.20	0.00	0.20	839.00	41.30	52.60
6	0.50	0.50	0.00	0.00	1009.00	54.55	80.46
7	0.50	0.40	0.00	0.10	999.00	51.68	76.90
8	0.50	0.30	0.00	0.20	989.00	48.80	70.40
9	0.50	0.20	0.00	0.30	979.00	45.93	46.24
High-volume POFA mix design							
10	0.00	0.30	0.00	0.70	1689.00	71.93	34.53
11	0.10	0.20	0.00	0.70	1539.00	64.43	23.04
12	0.00	0.40	0.00	0.60	1699.00	74.80	45.96
13	0.10	0.30	0.00	0.60	1549.00	67.30	37.80
14	0.20	0.20	0.00	0.60	1399.00	59.80	28.80
15	0.00	0.50	0.00	0.50	1709.00	77.68	55.64
16	0.10	0.40	0.00	0.50	1559.00	70.18	47.10
17	0.20	0.30	0.00	0.50	1409.00	62.68	40.60
18	0.30	0.20	0.00	0.50	1259.00	55.18	36.80
High-volume GBFS mix design							
19	0.30	0.70	0.00	0.00	1309.00	69.55	85.09
20	0.20	0.70	0.00	0.10	1449.00	74.18	97.75
21	0.10	0.70	0.00	0.20	1589.00	78.80	86.40
22	0.00	0.70	0.00	0.30	1729.00	83.43	70.53
23	0.40	0.60	0.00	0.00	1159.00	62.05	80.68
24	0.30	0.60	0.00	0.10	1299.00	66.68	72.44
25	0.20	0.60	0.00	0.20	1439.00	71.30	71.93
26	0.10	0.60	0.00	0.30	1579.00	75.93	70.84
27	0.00	0.60	0.00	0.40	1719.00	80.55	70.22
28	0.50	0.50	0.00	0.00	1009.00	54.55	80.46
29	0.40	0.50	0.00	0.10	1149.00	59.18	80.43
30	0.30	0.50	0.00	0.20	1289.00	63.80	67.22
31	0.20	0.50	0.00	0.30	1429.00	68.43	65.14
32	0.10	0.50	0.00	0.40	1569.00	73.05	56.34
33	0.00	0.50	0.00	0.50	1709.00	77.68	55.64
High-volume WCP mix design							
34	0.00	0.30	0.70	0.00	1323.81	58.66	34.02
35	0.10	0.20	0.70	0.00	1173.81	51.16	22.40
36	0.00	0.40	0.60	0.00	1385.98	63.43	68.44
37	0.10	0.30	0.60	0.00	1235.98	55.93	52.08
38	0.20	0.20	0.60	0.00	1085.98	48.43	46.76
39	0.00	0.50	0.50	0.00	1448.15	68.20	74.12
40	0.10	0.40	0.50	0.00	1298.15	60.70	66.19
41	0.20	0.30	0.50	0.00	1148.15	53.20	60.17
42	0.30	0.20	0.50	0.00	998.15	45.70	56.47
Average					1292.03	61.39	61.30
STDEV					293.71	12.26	18.70

**Table 4:** Characteristics of studied input and output parameters.

Parameters	Type	Unit	Max	Min	STD	Average
FA	Input	Mass (%)	0.70	0.00	0.21	0.25
GBFS	Input	Mass (%)	0.70	0.20	0.16	0.41
CWP	Input	Mass (%)	0.70	0.00	0.24	0.12
POFA	Input	Mass (%)	0.70	0.00	0.23	0.22
SiO_2_: Al_2_O_3_	Input	Ratio	8.63	2.10	1.58	4.03
CaO: SiO_2_	Input	Ratio	0.97	0.17	0.23	0.52
CaO: Al_2_O_3_	Input	Ratio	4.41	0.66	1.09	2.05
Age	Input	day	28.00	1.00	10.79	9.75
EE	Output	MJ/m^3^	1729.00	699.00	291.06	1292.03
CO_2_ emission	Output	kgCO_2_/m^3^	83.43	36.68	12.15	61.39

**Table 5:** Correlation matrix for determining input variables by PCA.

Parameter	Inputs							
	PCA 1	PCA 2	PCA 3	PCA 4	PCA 5	PCA 6	PCA 7	PCA 8
Eigenvalue	3.2321	2.3445	1.3253	1	0.0677	0.025	0.0055	0
Proportion	0.404	0.293	0.166	0.125	0.008	0.003	0.001	0
Cumulative	0.404	0.697	0.863	0.988	0.996	0.999	1	1

**Table 6:** Relationship between principal components and input variables.

Variable	Unit	PCA 1	PCA 2	PCA 3	PCA 4	PCA 5	PCA 6	PCA 7	PCA 8
FA	Mass (%)	−0.377	−0.336	−0.44	0	−0.521	0.113	0.122	0.502
GBFS	Mass (%)	0.349	−0.453	0.303	0	0.046	0.202	−0.628	0.379
CWP	Mass (%)	−0.241	0.307	0.667	0	−0.004	−0.11	0.271	0.563
POFA	Mass (%)	0.361	0.313	−0.503	0	0.46	−0.133	0.045	0.536
SiO_2_: Al_2_O_3_	Ratio	0.33	0.516	−0.007	0	−0.423	0.668	−0.008	0
CaO: SiO_2_	Ratio	0.374	−0.473	0.114	0	0.169	0.297	0.712	0
CaO: Al_2_O_3_	Ratio	0.547	−0.002	0.055	0	−0.554	−0.618	0.092	0
Age	Day	0	0	0	1	0	0	0	0

**Table 7:** Properties of cuckoo optimization algorithm (COA) parameters.

Parameter	Value	Parameter	Value
Number of initial populations	5	number of clusters that we want to make	1
Minimum number of eggs for each cuckoo	2	maximum number of cuckoos that can live at the same time	10
Max number of eggs for each cuckoo	10	Control parameter of egg laying	2
Max. iterations of Cuckoo Algorithm	300		

**Table 8:** Final weight and bias values of the optimum PCA-COA-ANN model.

***IW***					***b1***
0.2628	−1.693	0.5162	−1.0867		−2.0935
−1.3895	−1.4424	−0.1549	0.5895		1.0467
−1.045	−1.3043	0.1852	−1.2471		0
−0.9598	0.9747	−1.3313	−0.8596		−1.0467
1.3945	1.006	1.0623	0.5454		2.0935
***LW1***					***b2***
1.4828	−0.0699	0.045	0.7636	−0.7899	−1.8473
−1.2436	−0.1904	0.032	−0.3604	1.3034	0.6158
1.0932	−0.1355	0.8086	0.7932	0.9571	0.6158
1.1276	−0.792	1.2058	0.1342	0.2051	1.8473
***LW2***					***b3***
0.4349	−1.0378	−0.1033	1.2227		−1.6649
0.3141	−0.7172	−0.9725	−1.1014		1.6649

*IW*: Weights values for input layer; *LW1*: Weights values for first hidden layer; *LW*2: Weights values for the second hidden layer; *b1*: Bias values for the first hidden layer; *b2*: Bias values for the second hidden layer; *b3*: Bias values for the output layer.

## Data Availability

Data is contained within the article.
